# Transportability of confined field trial data for environmental risk assessment of genetically engineered plants: a conceptual framework

**DOI:** 10.1007/s11248-014-9785-0

**Published:** 2014-04-15

**Authors:** Monica Garcia-Alonso, Paul Hendley, Franz Bigler, Edgar Mayeregger, Ronald Parker, Clara Rubinstein, Emilio Satorre, Fernando Solari, Morven A. McLean

**Affiliations:** 1 Estel Consult Ltd., 5 Hillside Drive, Binfield, Berkshire, RG42 4HG UK; 2 Phasera Ltd., 7 Kenilworth Avenue, Bracknell, Berkshire, RG12 2JJ UK; 3 Agroscope Reckenholz-Tänikon, Reckenholzstrasse 191, 8046 Zurich, Switzerland; 4 Unidad de Gestión del Riesgo, Ministerio de Agricultura, Asunción, República del Paraguay; 5 Environmental Fate and Effects Division, Office of Pesticide Programs, United States Environmental Protection Agency, One Potomac Yard, 2777 S. Crystal Drive, Arlington, VA 22202 USA; 6 ILSI Argentina, Av Santa Fe 1145, 4° piso, C1059ABF Buenos Aires, Argentina; 7 IFEVA, Cátedra de Cerealicultura, Facultad de Agronomía y Veterinaria, Universidad de Buenos Aires, Avda. San Martín 4453, Buenos Aires, Argentina; 8 Monsanto Argentina SAIC, Estacion Experimental Fontezuela, Ruta 8 km 214, Fontezuela, Partido de Pergamino, Buenos Aires, Argentina; 9 Center for Environmental Risk Assessment, ILSI Research Foundation, 1156 Fifteenth Street NW, Suite 200, Washington, DC 20005 USA

**Keywords:** Genetically engineered plant, Confined field trial, Regulatory framework, Transportability, Agro-climatic zone

## Abstract

It is commonly held that confined field trials (CFTs) used to evaluate the potential adverse environmental impacts of a genetically engineered (GE) plant should be conducted in each country where cultivation is intended, even when relevant and potentially sufficient data are already available from studies conducted elsewhere. The acceptance of data generated in CFTs “out of country” can only be realized in practice if the agro-climatic zone where a CFT is conducted is demonstrably representative of the agro-climatic zones in those geographies to which the data will be transported. In an attempt to elaborate this idea, a multi-disciplinary Working Group of scientists collaborated to develop a conceptual framework and associated process that can be used by the regulated and regulatory communities to support transportability of CFT data for environmental risk assessment (ERA). As proposed here, application of the conceptual framework provides a scientifically defensible process for evaluating if existing CFT data from remote sites are relevant and/or sufficient for local ERAs. Additionally, it promotes a strategic approach to identifying CFT site locations so that field data will be transportable from one regulatory jurisdiction to another. Application of the framework and process should be particularly beneficial to public sector product developers and small enterprises that develop innovative GE events but cannot afford to replicate redundant CFTs, and to regulatory authorities seeking to improve the deployment of limited institutional resources.

## Introduction

The development of a genetically engineered (GE) crop plant follows a progression from experimentation in laboratory and other contained facilities, to field studies, and eventually to cultivation after pre-market environmental risk and food/feed safety assessments have been conducted by the appropriate regulatory authorities. Conducting field studies with experimental GE plants, termed confined field trials (CFTs), is a regulated activity, meaning that permission must be obtained from the appropriate competent authorities before trials can be planted. Permitted CFTs are performed under a regime of management practices designed to confine the trials so as to prevent the accidental release of plant material from the trial site, trait introgression into populations of sexually compatible species, or establishment of populations of the experimental GE plant in the environment (see examples such as CFIA 2011b; CLI 2010; OECD 1992).

CFTs are regarded as essential for the evaluation of regulated GE plants under realistic environmental conditions and hence are conducted in agro-ecosystems (also referred to as receiving environments) representative of those where that particular GE crop may be cultivated. Typically, and in accordance with internationally accepted approaches to environmental risk assessment (ERA) of GE plants (OECD 1992; SCBD 2000), a comparative assessment is followed where the GE plant is compared to its conventional counterpart, usually the isogenic or a near-isogenic line, which is included in the CFT as a control. Trial endpoints vary depending on the risk hypothesis being tested, but most CFTs aim at identifying any differences between the GE event and its non-GE comparator resulting from intended or unintended consequences of the genetic modification across a range of agro-ecosystems (OECD 1992; SCBD 2000). Design of CFTs is optimized to obtain data relevant to risk hypotheses while minimizing confounding factors that may interfere with the comparison, for example damage by biological stressors such as weeds, pests and diseases.

There are no international standards for conducting CFTs, and national regulations and guidance vary by country in details about trial design, location and duration as discussed in more depth below. However, it is commonly held that field studies used to evaluate the potential environmental risks associated with a GE plant that is being considered for cultivation approval should be conducted in each country where cultivation is intended, and some countries explicitly require in-country CFTs for any GE event that will be submitted for cultivation approval (e.g. CTNBio 2008; MoE 2004). This means that multi-site CFTs are often repeated on a country-by-country basis, irrespective of any similarities between growing environments. Since CFTs are regulated, their conduct requires substantial financial, institutional and human resource investments by both regulatory authorities and product developers, and this represents a significant regulatory burden (Nuffield Council on Bioethics 2003). Requirements to conduct duplicative CFTs that provide no additional, informative data for use in ERA is particularly challenging for public sector and small enterprises working with limited resources.

It seems reasonable that data from CFTs conducted in one country (henceforth referred to as the “remote country”) could potentially be accepted as relevant, and even sufficient, for the purposes of ERA by regulators in another country (henceforth referred to as the “local country”) i.e., that CFT data relevant to ERA should be transportable between countries. This concept can be readily applied in practice if the agro-climatic zone where a CFT is conducted is demonstrably representative of the agro-climatic zones in those geographies to which the data will be transported. In an attempt to elaborate this idea, a multi-disciplinary Working Group of scientists collaborated to develop a conceptual framework and associated process that can be used by the regulated and regulatory communities to support transportability of CFT data for ERA.

The goal of this paper is to provide an outline of the resulting conceptual framework and recommend some enabling steps that should be followed to provide the necessary scientific support to build confidence in data transportability. The Working Group believes that developers of GE plants (including governmental and academic research institutions, and both large and small private enterprises) should carefully consider how a framework such as this can be applied to select highly relevant locations for CFTs, and to design such trials to maximize opportunities for data transportability. The framework additionally provides regulatory authorities and risk assessors with an objective, evidence-based rationale and supporting process that, if applied, should further the acceptance of remote data and permit regulators to evaluate if remote data alone may be sufficient to complete a local ERA. Several potential scenarios illustrate how this approach could improve data transportability and it is anticipated that follow-up publications will provide examples of alternative ways of approaching the detail behind the overall concepts.

## Confined field trials and the regulatory process

In most countries the regulatory system for approving the cultivation of GE crops requires the preparation of an ERA to facilitate decision making. The first step in such an ERA is usually problem formulation, where policy-derived protection goals and the scope of the risk assessment are taken into account (Raybould 2007; Sanvido et al. 2011; Wolt et al. 2010). Those involved in the ERA process compile relevant information to establish whether there are sufficient data to complete a risk characterization. The information typically considered during the problem formulation step is gathered from various sources, such as peer reviewed scientific papers, scientific opinions generated by regulatory authorities, data generated to support food/feed safety assessment, and data generated for the same GE event in other geographies. Following an analysis of these data, risk assessors can determine if further information is necessary and whether or not additional data are required (Garcia-Alonso 2010). Where the outcome of problem formulation indicates that CFTs are necessary to address specific risk hypotheses, then trial design, the number and locations of trials, and key measurement endpoints are identified.

CFTs are designed to compare specific endpoints between the GE plant and its conventional counterpart(s) under the same climatic and agronomic conditions. Given the natural variation associated with growth processes and field studies, careful replication and data analyses are needed to account for variations in mean endpoint values. The usual design of CFTs involves replicated test and control plants in randomized blocks. Data collected from reference varieties included in the study design, or historical data from previous trials with common commercial cultivars, can be used to provide a context for the interpretation of any observed or measured differences that may be due to natural variability.

In order to maximize the likelihood of detecting any actual differences between GE and non-GE endpoints, CFTs are managed using local agronomic practices typical for that crop. These practices include the use of fertilizers, irrigation, tillage, and maintenance chemicals (e.g., pesticides and/or herbicides) as appropriate to ensure the production of a successful crop. The routine application of weed, disease and insect control measures also helps to reduce variability between trial site locations with respect to biotic stressors. Experimental evidence consistently shows that differences between locations, years, genetic backgrounds and agronomic practices contribute more to endpoint variation than the process of transgenesis (Harrigan et al. 2010; Ricroch 2012), so differences in endpoint measurements are often detected between different varieties of the same crop planted under very different conditions, but not between the GE and its non-GE counterpart grown under similar conditions.

Many regulatory authorities have published guidance that prescribes information and data requirements for ERA that are considered on a case-by-case basis for each GE event (ideally, during problem formulation). Included in this guidance may be criteria related to the purpose and/or design of CFTs (Table 1). In the context of ERA for cultivation approvals, CFTs are typically used for at least three purposes: (1) to generate plant material (e.g., grain and/or forage as appropriate to the crop uses) for compositional analyses; (2) to collect samples of various plant tissues (e.g., leaf, root, stem, pollen, forage, grain) at different stages of plant development to quantify levels of expressed novel protein(s); and (3) to collect relevant phenotypic and agronomic data over the lifecycle of the plant. It is common for a CFT to be designed to simultaneously meet two or all three of these purposes.

**Table 1:** Example design criteria for confined field trials of genetically engineered plants

Country/region	Criteria	Reference
Canada	Max. 1 ha/trial site; 5 ha total/submission/province. Max. 10 locations/province/submission. Min. 2 years/event recommended	CFIA (2010, 2011b)
European Union	CFTs for compositional analyses and agronomic comparisons in eight locations, min. 1 year CFTs for expression data in three locations, min. 1 year.	EFSA (2011)
India	Biosafety Research Level I trials, max. trial size 0.4 ha, min. 2 years Biosafety Research Level II trials, max. trial size 1 ha, max. eight trials/event, min. 1 year	DBT (2008)
United States	Recommended parameters for corn (minimum 8 sites) and cotton (minimum 6 sites over 2 years or 12 sites over 1 year); not prescribed for other plant species. Multi-year testing preferred but not required	USDA (2007, 2012, 2013)
Argentina	Not prescribed.	MAGy (2011)
Australia	Not prescribed.	OGTR (2009)
Brazil	Not prescribed.	CTNBio (2008)

The authors recognize that CFTs are also conducted for other purposes, such as for basic research, to produce sufficient plant material for analytical or feeding studies applicable to food/feed safety assessment, for event selection, and for variety registration. However, the conceptual framework developed in this paper is purposefully limited to considering those CFTs designed to provide data that are relevant to evaluating the potential adverse environmental impacts of a specific GE event that is being considered for cultivation.

### CFTs for compositional studies

Compositional equivalence testing that compares concentrations of key components (e.g., nutrients and anti-nutrients) of the GE plant with the same in the conventional counterpart is a key element of the safety assessment of GE foods/feeds (CAC 2003). Compositional data generated from CFTs conducted out-of-country are already readily accepted by regulatory authorities for the safety assessment of imported GE food/feed. Countries intending to import GE food or feed need to establish whether the products will be safe for humans and animals, and because the GE event from which the food/feed is derived is not cultivated locally, compositional data from CFTs in the country of cultivation are accepted. However, when it comes to ERA for cultivation approvals, the usual practice is for local data generation even when compositional data from remote CFTs are available. There are examples where remote data alone were considered sufficient (e.g., CFIA 2007, 2008, 2011a), and there are regulatory authorities that foresee the feasibility of accepting this approach if justification is provided that the agro-climatic zones where remote CFTs are conducted are representative of the conditions of cultivation of the GE crop locally (e.g. EFSA 2010).

### CFTs for expression data

Fundamental to the exposure characterization in an ERA is being able to establish the expected environmental concentration of the transgenic protein (or other novel molecule) expressed in the GE event (USEPA 1998, 2007; Wolt et al. 2010). Typically, exposure assessment requires data on levels of novel protein expression in different plant tissues at different life stages of the plant (e.g. CFIA 1998, 2001, 2012; CTNBio 2008; EFSA 2010, 2011). GE events may have persistent, limited or no expression of the transgenic protein in specific tissues or the level of protein expression may change in specific tissues over time (CERA 2011a, c, b; d and references therein; Park et al. 2010). Expression data are therefore used both to estimate how much of the transgenic protein(s) a particular organism might be exposed to under natural conditions, as well as potential persistence and/or accumulation over time.

### CFTs for agronomic and performance data

The phenotypic parameters measured in CFTs are crop-specific and generally encompass those characteristics relevant to plant emergence and vegetative growth (e.g., germination, early stand count, seedling vigor, plant height, ear height in the case of maize, lodging, final stand count) as well as those related to reproductive biology of the plant (e.g., time to silking in the case of maize, time to flowering or pollen shed, pollen viability, time to maturity, yield, and extent of pod shattering in the case of soybeans or canola). For those plant species where there is a long history of conducting CFTs (e.g., maize, soybean, cotton and canola), there is some standardization in the phenotypic characteristics that are evaluated which helps facilitate data transportability.

### CFTs for other purposes relevant to ERA

Problem formulation may identify additional risk hypotheses that are best addressed through field testing. Examples could include: insect-resistant events where early tier testing of the insecticidal protein (or other novel active ingredient) for adverse effects on non-target organisms indicates that higher tier (field) testing is warranted (Carstens et al. 2012; Duan et al. 2010; Romeis et al. 2008, 2011); or GE plants that express a trait that will permit cultivation of the plant species outside its normal geographic range. In such cases, it is likely that local CFT data will be necessary to complete the ERA.

## Conditions for CFT data transportability

The Working Group considered what conditions would need to be satisfied for a local regulator to accept remotely developed CFT data for the purposes of an ERA for a cultivation approval. Four key conditions were identified:

- The CFTs must have been conducted, and data documented and reported, in a manner that meets minimum local regulatory requirements;
- The environmental and agronomic conditions under which the CFT was conducted in the remote country(ies) must be relevant to the conditions in the local country where the GE event is intended to be cultivated;
- The local regulator would need to be provided with an evidence-based justification for accepting data from CFTs conducted in the remote country(ies);
- The local regulator would need a science-based process for identifying whether CFT data developed in one or more remote countries are sufficient to address local needs, or whether additional trials might be required.

This paper attempts to provide a framework that will allow conditions A-C to be met. Approaches and next steps for addressing condition D are included in the Discussion.

The authors also considered that for the framework to be workable, it should:

- Avoid making the existing ERA process more complex than necessary;
- Whenever possible, leverage existing agronomic expertise and established methodologies to provide scientific support for data transportability but also suggest approaches to address cases where existing data do not provide sufficient information;
- Continue use of existing CFT study designs;
- Use historical data for *post facto* validation of the proposed framework/process;
- Ensure any data or technologies necessary to support regulatory use of the framework are “open” i.e., are publicly available, free or affordable, and fully documented to robust metadata standards (G8 2013).

The Working Group focused on the particular needs of two key groups of stakeholders when identifying the criteria that must be addressed for data to be transportable. The first are the data developers, a class which includes public and private sector product developers. They design and conduct CFTs to meet the data requirements of regulatory authorities in those countries where cultivation of GE events are intended. Important drivers for this group of stakeholders are the time and costs involved in obtaining regulatory authorizations so that products can be deployed as efficiently as possible. The second set of key stakeholders are the regulators who are responsible for ensuring that risk assessments and subsequent product-specific decision-making are based on sound science and are conducted in accordance with national and international obligations. This group must ensure that the outcome of each risk assessment is scientifically defensible, and that decision-making is transparent so that expectations as regards predictability and accountability can be achieved. Both of these groups should benefit from overall workload reductions if data from CFTs in remote countries can serve to address ERA requirements in a local country. As will be seen later, precedents for the mutual acceptability of field studies conducted in different countries have been established in other heavily regulated arenas *e.g*. pesticide environmental exposure assessments. The concepts proposed here are only an extension of regulatory policies and practices already in use by other government agencies involved in ERA.

## Addressing condition A through development of minimum CFT design and reporting guidance

The types of CFTs and the endpoints measured in each depend upon the risk hypotheses developed in the problem formulation step and/or data requirements prescribed in regulations and/or guidance. As described above, and unlike other related regulatory arenas in agriculture such as pesticide testing (see Box 1; OECD 2013), there is little internationally accepted guidance that specifies minimum CFT design and reporting requirements. If such documentation existed, it would be simpler for regulators to evaluate the acceptability of trial data developed in a remote country i.e., to efficiently review the data and to compare and contrast different studies. Moreover, the standardization of study design and acceptability criteria can open the way to global regulatory review as has occurred with the OECD Council Acts in 1981 and 1989 supporting the Mutual Acceptance of Data in the area of chemical testing (OECD undated). Harmonized CFT guidance that focuses on specifying only the essential and minimum set of requirements is unlikely to increase (and may even reduce) regulatory costs.

**Box 1:** Transportability of field data for ecological risk assessment of pesticides

As the concept of transportability of CFT data is largely aimed at addressing regulatory requirements associated with ERA submissions for cultivation approvals in different countries, it is realistic to explore whether there are precedents for similar approaches in other related regulatory arenas. One such example is in the multi-national risk assessment of conventional pesticides where common approaches to classification, comparison and grouping of soil types are already used for various regulatory laboratory and field studies. Soils are selected in such a way that field tests performed in one region produce results that are valid for use in pesticide risk assessments in other regions of the world. This process is a result of enhanced cooperation between countries and has in turn led to a more efficient and productive system of global pesticide reviews. The use of field studies conducted at foreign sites for national and global joint reviews reduces economic and regulatory burdens for both registrants and regulators. This approach depends upon the existence of trans-national soil classification schemes which have been developed to provide scientists and resource managers with generalized information about the nature of a soil found in a particular location. The soil classification schemes are analogous to the agro-climatic zones for plant growth; environments that share comparable soil forming factors produce similar types of soils globally. The United States Environmental Protection Agency pesticide test guidelines for environmental fate, transport, and transformation state that "test soils used in these studies should be collected from typical, intended pesticide use areas in the United States" and that "soils from foreign sources may also be used in conducting these fate studies if the foreign soil has the same characteristics as a soil in the United States from a similar use area. Furthermore, complete information on the soil class, textural characterization, pH, organic matter content, and soil classification should be provided by the pesticide registrant so that EPA can determine if the chosen soil is representative of agricultural soils that are found in the US" (USEPA 2011). Information related to classification of foreign soils is critical in evaluating selected environmental fate studies and their spatial relevance to soils where a pesticide is proposed to be used. The Ecoregion Crosswalk Similarity Model has been developed to maximize the use of pesticide field dissipation studies by developiing harmonized international guidance for conducting the studies and identifying comparable North American and European Ecoregions (OECD 2012). This geospatial tool is essentially a GIS-based Decision Support System which can identify comparable North American and European Ecoregions (e.g., Bailey 1996; Liu and Samal 2002; Omernik 1993; Waltman et al. 1999) in order to assist the pesticide industry and regulatory authorities in the selection of regions for field study sites, and provide background information on pesticide use areas (crop-based), soils and climate including location.	

This Working Group recommends that CFT study reports should provide sufficient information to demonstrate that a trial was conducted in a given agro-climatic zone and experienced conditions representative of the agronomic and climatic conditions characteristic of that zone since this is essential if a regulator is to be able to consider the data from a remote CFT as relevant to the local situation. The report should also detail the design of the trial and the endpoints measured, the agronomic practices used, the climatic conditions encountered during the trial, and any unusual observations regarding pest and diseases. Accordingly, in addition to all necessary data on endpoint measurements and statistical comparisons, the data which should be available include:

- Location data for the CFT *e.g*., latitude, longitude, Global Observing System location and elevation;
- Local weather data at the trial site for the duration of the CFT, including at least daily temperature and precipitation (ideally on-site), solar radiation, and day length (if necessary, from a nearby weather recording station);
- Historical weather data from the closest long term high quality weather recording station. Ideally this should span a period of at least 20 years and should include recent years to account for modern climatic conditions. While daily data specify the actual growing conditions for the particular year of the CFT, 20 year historical data are useful to characterize the climate of the general area where the data were generated. Moreover, a 20 year historical database provides enough information to put the actual CFT conditions into climatic context opposite expected weather patterns using statistical comparisons or simulation models;
- Details of the trial including:
  - Planting, crop development, maturity and harvest dates;
  - Soil preparation and nutrient supply;
  - Physical and biological stress management operations (e.g. pest control, irrigation);
  - Other agronomic practices.
- Trial specific anomalies (*e.g*., exceptional water deficit or surplus periods, frosting, pest attacks).
- Any issues dependent upon the nature of the GE event where the trait might impact plant response to unmanaged natural conditions (e.g. abiotic stress tolerance traits).

Provided with these data, a risk assessor can make an informed determination as to whether a trial has been well conducted and whether endpoint information from a CFT conducted in a particular agro-climatic zone in one or more remote countries is relevant to the agro-climatic zones in the local country where the GE event is proposed for cultivation.

## Addressing condition B by demonstrating the relevance of CFT conditions in a remote country to the local country

The Working Group spent considerable time examining those biotic and abiotic factors that influence crop production in CFTs in different locations; unsurprisingly, it appears that the physical environment (climate, weather conditions, soil etc.) and cultural practices are the most significant. As discussed above, CFTs are managed to control biotic factors that can potentially affect the comparison of endpoints, and therefore were not considered by the Working Group to be as defining as the physical characteristics of the trial site which were subsequently categorized as follows:

1. The impact of CFT trial management on the measured endpoints;
2. The spatial distribution of cultivation of a given crop at global, regional and local scales;
3. The climatic variables that define growth and yield of crops.

### The impact of CFT trial management on the measured endpoints

As previously described, CFTs are used to compare the same endpoints for the GE event and its conventional counterpart(s) and so are typically designed to control for confounding factors. Examples include:

- Soil texture and tillage—the plots for GE and non-GE plants are planted, as far as possible, in plots with homogeneous soils that have been managed in the same way and which are well known to be locally suited to production of the crop of interest;
- Nutrient status—as far as possible the GE and non-GE plots receive uniform nutrient inputs at identical timings;
- Control of pests and other biological stressors—although this depends to some extent on the trait introduced, biotic stressors are controlled using agronomic interventions typical for that crop species, such as application of pesticides;
- Cultural practices including time of planting, agronomic management etc., are effectively identical between GM and non-GM plots at the same trial site.

These statements help define the minimum set of data required to report a CFT in a format that will readily support data transportability (see Condition A. above).

As a result of these CFT management practices, several potential sources of variation (both within a trial location and between trial locations) are reduced or removed. As a result, a working assumption can be made that the key variables that categorize the physical conditions under which a CFT is conducted can be reduced to the overall weather pattern experienced during the trial period and whether any unusual, uncontrolled phenomena occurred that might have led to unanticipated differences between GE and non GE plots (e.g., severe pest pressure, severe drought).

### The spatial distribution of commercial production of a given crop at global, regional and local scales

A fundamental and obvious statement that applies to most of the major world food and fiber crops is that the current global cropping patterns provide strong evidence of the relatively narrow spatial range over which at least some crops and their varieties can be grown (Bunting et al. 1982; Kassam et al. 1977; Loomis and Connor 1992). Since Klages (1942), many authors have looked at the relationships among climate, soil and biotic factors, and the distribution and production of crops. Moreover, global and regional cropping systems maps are abundant and have been available for decades (FAO 1999; Fischer et al. 2002). Despite the fact that social (*e.g*., cultural and historical factors) and evolutionary factors (e.g., Jennings and Cock 1977; Viglizzo 2001) have some influence on crop distribution, ecological studies (see references above) widely accept that climate, soil and crop management techniques are key determinants of the successful growth of a species in any location. A CIMMYT review provides examples of “maize mega-environments” that exemplify this concept (Hartkamp et al. 2000).

**Fig. 1:**
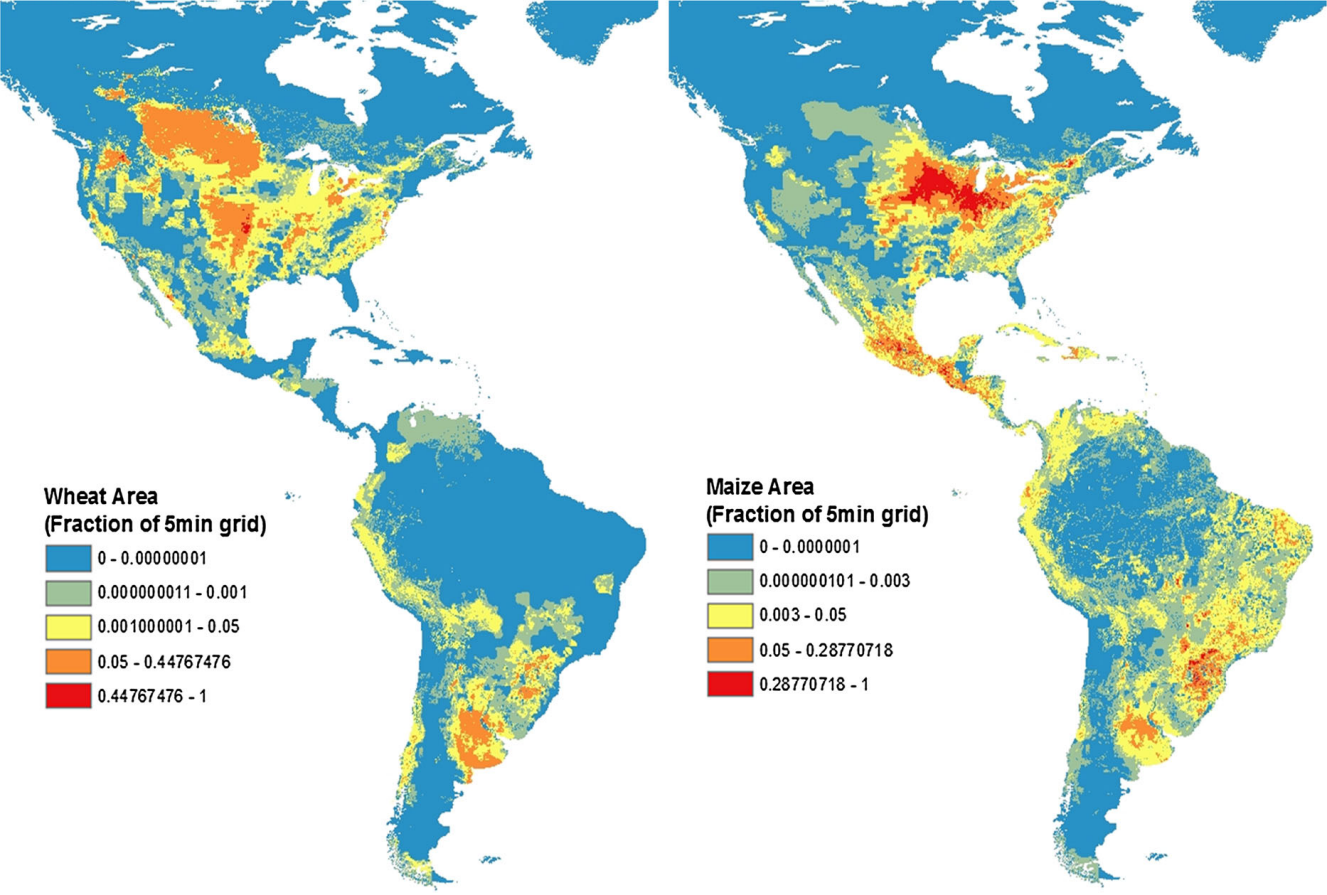
Crop planting area maps for North, Central and South America exemplifying crop-specific agro climatic distributional differences for wheat and maize. Spatial data downloaded from http://www.sage.wisc.edu as described in Monfreda et al. 2008. Area expressed as the fraction of crop of interest in a five arc-minute grid (each approx.10 km by 10 km)

From the point of view of CFT data transportability, current global crop maps provide an immediate indication of the range of key growth variables that the crop can tolerate and still be reasonably productive. Figure 1 shows examples of crop distribution maps for wheat and maize. Undoubtedly, the clustering of species by latitude in the northern and southern hemispheres greatly depends on climatic factors influencing growth and development of both crops. Although widely distributed, wheat and maize have exploited relatively narrow ranges of agro-climatic conditions.

### The climatic variables which define growth and yield of crops

Climatic and soil constraints regulate the performance of crops and hence their management in any region. Crop simulation models have become a valuable tool to explore the effects of these factors on yield and, more recently, spatially-explicit tools have been developed allowing the comparison and evaluation of yield under geographical references. Intensive research and development of crop growth models together with rapidly expanding spatial tools and data sets have allowed the possibility of identifying “climate analogues”. An example of the strength of these approaches is found in Ramirez-Villegas et al. (2011) where the authors explored the concept in terms of answering several questions including:

- Where can I find sites that are analogous to my selected site?
- Which sites may be like my selected site at some point in the future?
- Which sites may have been like my selected site in the past?

In other words, they examined the concept of spatially analogous sites where the climates are currently similar as well as temporal analogs where the climate may be expected to be similar at some other point in time. Ramirez-Villegas et al. (2011) developed spatio-mathematical approaches to examine dissimilarity and uncertainty measures for a wide range of bioclimatic variables. Thus it is clear that tools are available that permit the development of well-defined metrics to quantify how similar the climate in one particular region is to that of other regions. Two examples of how these tools can be applied are provided below.

In a detailed analysis of maize cropping, Solari et al. (2012) used yield data from field trials of hybrid maize along with long term weather data to identify similar agro-ecozones in South America. Combinations of agronomic and climatic variables provided a wide range of environmental conditions under which growth and yield performance of various maize genotypes were evaluated. A multivariate cluster analysis approach on weather variables was used and the analysis showed that only a few variables were necessary to identify similar crop climatic zones; regional climatic clusters could be distinguished by temperature, rainfall, air relative humidity, growing degree days, elevation, and day length (Solari et al. 2012). Analysis of historical weather data indicate that North West Argentina clearly clustered with some parts of Brazil and subtropical Mexico. Clustering with Brazil was also supported by genotype + genotype by environment (GGE) analysis and rank correlations performed on 37 hybrids during 2006, 2007 and 2008 growing season (Solari 2010). Using GGE and rank correlation, locations in Argentina and Paraguay grouped closer to Brazilian locations than to other Argentinian locations. As a result, they proposed the use of locations in North West Argentina as surrogate testing environments for tropical and subtropical corn typically grown in Mexico and Brazil. Similarly, Menéndez and Satorre (2007) found that radiation, a photo-thermal quotient (Savin and Slafer 1991), and temperature during grain filling explained most of wheat potential yield variability within the Argentine Pampas. These examples support the idea that a few climatic variables may be useful to identify similar geographical patterns in relation to crop yields and performance using existing tools, and therefore are relevant factors to be recorded when conducting CFTs.

### Identification of critical agro-climatic factors and crop specific agro-climatic zones

The Working Group recognized that, while potentially satisfactory, unique approaches along the lines discussed above do not meet the goal of providing an acceptable and accessible science-based methodology suitable for accepting data for regulatory decision-making. As a result, the Working Group investigated approaches available in the peer reviewed literature that can be applied to do this. One of the methodologies examined was the concept of agro-climatic zones (ACZ) and agro-ecological zones (AEZ). These are approaches for dividing a region based on either homogeneity between the weather parameters which are the most significant determinants of crop growth and yield (in the case of ACZ) or are based on combinations of weather and soil factors (for AEZ). These can either be crop specific, in which case they divide the regional or global crops maps (as in Fig. 1) into sub-divisions based on ACZ or AEZ, or they can be independent of existing crop production regions and may then reflect conditions across the total land area or just across those areas on which there is agricultural production. A recent review based on the need to better simulate crop yield gaps has compared many of the currently accepted zonal approaches (van Wart et al. 2013). This analysis demonstrated that there are currently existing zonation schemes that have received peer review scrutiny and that are already being used for extrapolating point data (analogous to CFTs conducted in a remote country) to areas with similar agro-climatic conditions elsewhere in a region and/or globally (see Box 2).

**Box 2:** An evaluation of approaches for establishing agro-climatic zones and agro-ecological zones

In order to determine if existing zonation schemes developed for other purposes could be applied to extrapolating CFT data from local to remote countries, the Working Group considered the six zonation schemes described in van Wart et al. (2013) and summarized in Table 2 below. Based on the working assumption that CFT design effectively removes soil related factors from further consideration (since the trial is comparative), the Working Group’s evaluation focused on agro-climatic rather than agro-ecological zonation approaches. As a result, the SAGE, GAEZ_LGP and HCAEZ approaches were discounted from further consideration by the Working Group. The van Wart et al. (2013) analysis indicates that the three remaining schemes also have markedly smaller average zone areas (similar to SAGE); as a result, the zones tend to have quite narrow ranges of temperature and water availability. The GEnS and GYGA-ED approaches also have the smallest ranges of precipitation and temperature seasonality. As van Wart et al. point out, there is a difficult balance to be struck between having well delineated agro-climatic zones and minimizing the numbers of zones needed to define a given crop. Given the fact that SAGE relies on soil data, the Working Group determined that the optimum trade-off between these two constraints seems to be offered by the GeNS and GYGA-ED approaches, with GEnS requiring a total of 30 zones to address 80 % of the global rain fed maize production while 5 and 13 zones respectively define 80 % of US and Chinese maize production respectively. For transportability of CFT data, it remains to be determined if the optimal zonation approach should be: crop specific (SAGE and GLI); develop a uniform set of zones for all crops (GEnS); or consider uniform agro-climatic variables but only for grid cells representing areas where major food crops are known to be grown.

**Table 2:** Existing agro-climatic and agro-ecological zoning approaches (from van Wart et al. 2013)

ACZ/AEZ scheme	No. of zones	Type of AEZ	Variables considered, methodology	Reference
GAEZ-LGP^a^	16	Matrix	Temperature, precipitation, potential evapotranspiration and soil characteristics are used to calculate length of growing season	Fischer et al. (2012)
HCAEZ^b^	21	Matrix	Mean temperatures, elevation, and GAEZ-LGP are used to define thermal regimes and temperature seasonality	Wood et al. (2010)
SAGE^c^	100	Matrix	Growing degree days (GDD; Σ Tmean–crop-specific base temperature) and soil moisture index (actual evapotranspiration divided by potential evapotranspiration).	Licker et al. (2010)
GLI^d^	25	Matrix	Harvested area of target crop, crop-specific GDD and soil moisture index (actual evapotranspiration divided by potential evapotranspiration).	Mueller et al. (2012)
GEnS^e^	115	Cluster	Four variables (GDD with base temperature of 0 °C, an aridity index, evapotranspiration seasonality, temperature seasonality) used in iso-cluster analysis to “cluster” grid-cells into zones of similarity.	Metzger et al. (2013)
GYGA-ED^f^	300/265	Hybrid	Hybrid of GLI/GEnS schemes above—focus on grid cells with >0.5 % of area a major food crop. Does not require soil data	GYGA (2013)

^a^ Global agro-ecological zone length of growing period

^b^ Harvest choice agro-ecological zone

^e^ Center for sustainability and the global environment

^d^ Global land initiative

^e^ Global environmental stratification

^f^ Global yield gap atlas extrapolation domain

### Concept for an equivalent case-specific methodology for generating agro-climatic zones where needed

The previous section describes the application of the proposed framework for CFT data transportability using available peer-reviewed zonation schemes. However, the Working Group also developed an alternative approach that can be applied on a case-specific basis either as a verification/validation of the ACZ approach outlined above, or as a tool for those rare cases where standard ACZs would not be applicable (e.g., when dealing with crops for which no publically-available crop specific ACZs exist or when examining a GE plant expressing a trait which will allow the plant species to be grown outside of its current range of cultivation).

**Fig. 2:**
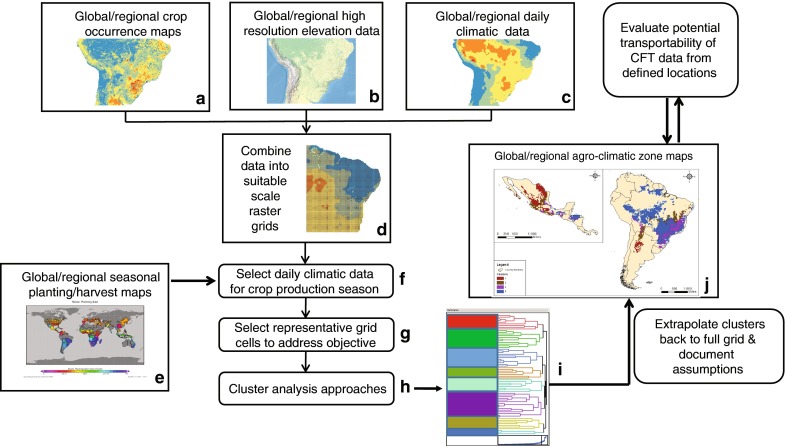
Outline of the alternative case-specific methodology for generating agro-climatic zones

This alternative approach is presented in Fig. 2. In outline, it requires developing a conceptual model of the key climatic and geophysical parameters that will adequately define the regional or global conditions needed for successful cultivation of the GE plant. Typically this will involve best professional judgment based on literature data and knowledge of the plant species involved. Daily weather data sets containing these parameters need to be accumulated and allocated across a convenient grid base along with any necessary geophysical data (represented by Fig. 2a, b, c) being combined into a raster GIS (Fig. 2d). This spatial database is then queried for appropriate crop growing periods based on best professional judgment and available data on global and regional planting and harvest dates, represented by Fig. 2e. One complication here is any necessary adjustment for different seasons in areas where the weather conditions allow for serial cropping of a single plant species. This process generates a gridded set of climatic data applicable to the particular growing period(s) for the GE plant at a global or regional scale (Fig. 2f). The next task is to subsample the gridded map to identify a representative number of locations reflecting the entire expected cropping area in the region (or globally). The selected cells should be ones where cultivation of the crop is known to occur or for GE events aimed at extending the geographic range of cultivation, areas for future crop deployment. To maximize applicability for CFT data transportability, these example grid cells should ideally include both the remote and local countries and, where known, the location of the remote country CFTs (Fig. 2g). This generates a representative set of daily climatic data describing the appropriate regional cropping seasons in locations known for cultivation of the crop of interest. This set can then be subjected to cluster analysis (Fig. 2h) to divide the representative subset of cells into a realistic set of discreet agro-climatic clusters. Extrapolation of the clusters back to the full GIS database with accompanying “lumping” into homogeneous discreet zones allows the creation of a case-specific set of agro-climatic zones which may be used in the same way as the publically available ACZs described above (Fig. 2i, j).

## Addressing condition C through application of a conceptual framework for data transportability

The steps above have identified the necessary report content required to fully characterize a minimum set of CFT data which would meet local regulatory requirements and would also provide the necessary information to demonstrate that the remote CFT was conducted in a specific agro-climatic zone and experienced conditions during the trial representative of the agronomic and climatic conditions characteristic of that zone. In addition, the Working Group has identified that given the within-trial comparative design of CFTs, the key variables investigated across a range of CFTs are climatic. Several existing peer-reviewed schemes have been reviewed for identifying global agro-climatic zones—either crop specific or for general agricultural production; these schemes have unique characteristics but several appear to meet the needs for characterizing similarities between cropping regions in different countries. By drawing these aspects together, a framework for CFT data transportability was developed.

The goal was to develop an approach to determine if the environmental conditions at CFT sites in a remote country (or countries) are relevant to the areas of the local country where the GE event under consideration for cultivation will be deployed. The resulting process should make use of acknowledged agronomic expertise and established methodologies where possible, and be recognized as providing a science-based rationale suitable for accepting data for regulatory decision-making. However, in those cases where pre-existing data/approaches are not available, the same concept should be amenable to case (crop)-specific generation of information for comparing environmental conditions between remote and local agro-climatic zones.

The framework is simple, with the following logic flow:

1. Do the data from the CFT(s) conducted in a remote country meet the local regulatory requirements?
2. Was the CFT conduct and in-trial weather conditions free of anomalies that might make it questionable for local use?
3. Can the particular agro-climatic zone in which the CFT was conducted in the remote country be identified?
4. Is that agro-climatic zone also relevant to crop production in the local country?

**Fig. 3:**
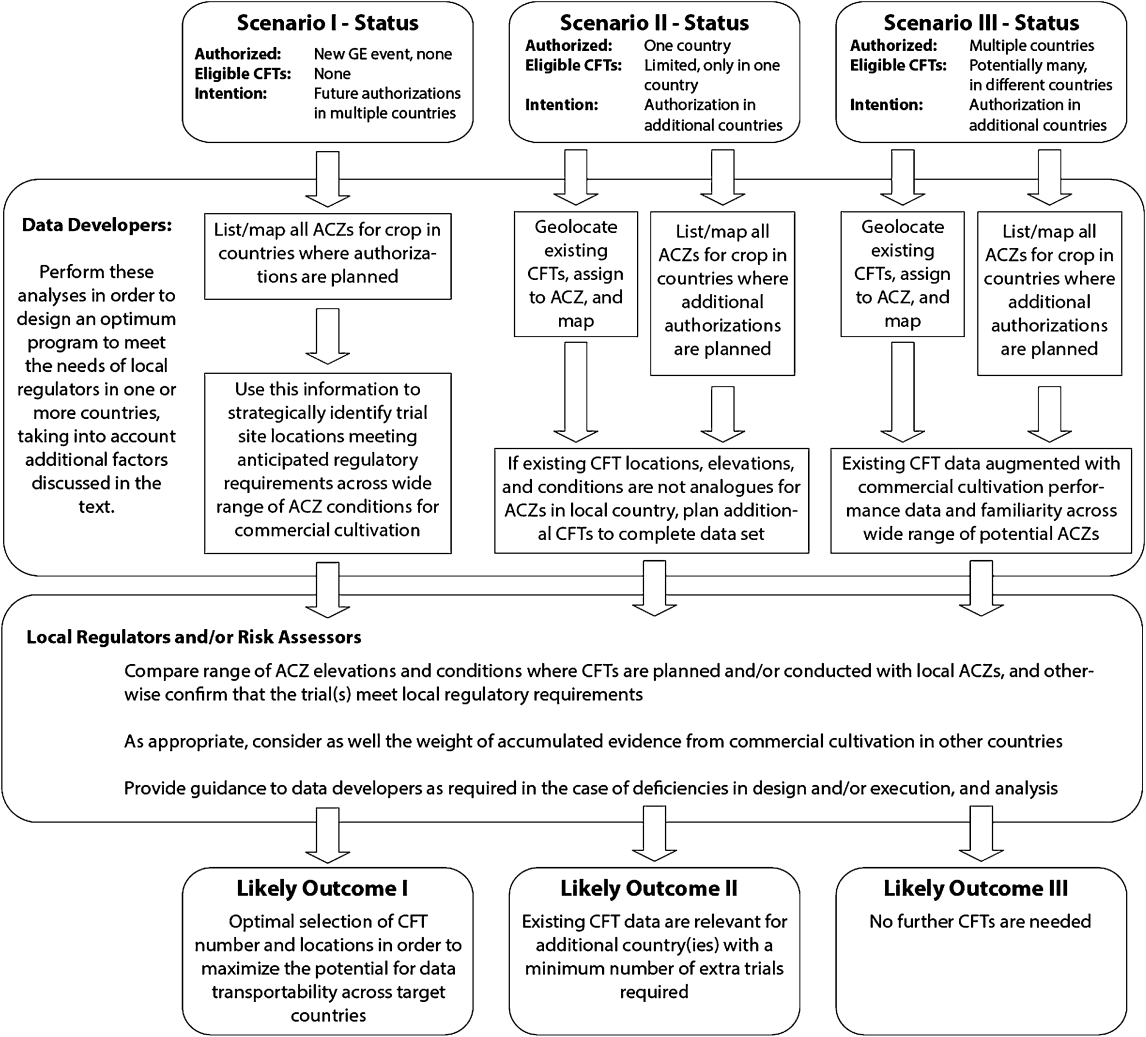
Flow chart for a process for applying the conceptual framework to enable transportability of CFT data for ERA (*ACZ* Agro-climatic zone, *CFT* confined field trial)

If the answers to those questions are all positive, then the CFT trial data should be eligible for regulatory use in the local country. However, in the real world, regulatory decision making is not quite so straight-forward and so the Working Group has refined the framework to present its potential application in the context of three scenarios, and from the perspectives of the two key stakeholder groups, summarized in Fig. 3 and described in more detail below.

*Scenario I: A GE event that has yet to be approved for cultivation in any country and will be evaluated in CFTs for the first time.*

In this case, the product developer can use the framework to strategically identify trial site locations that will be representative of the range of (global or regional) agro-climatic zones where the event may eventually be cultivated. By using crop-specific agro-climatic zone maps, careful site selection and trial design should permit the collection of data that will be relevant to different receiving environments and that will meet the specific requirements of those regulatory systems where approvals for cultivation will be sought. Depending on whether the GE event has a trait of limited or broad geographic utility, representative trial sites might be found in a single country or, more likely, locations may have to be identified across multiple countries. Other considerations that may affect where to locate CFTs include:

- Ability to obtain permission from regulatory authorities to conduct the trials;
- Capacity to ensure management and control of the trial sites for the duration of the trial and during any period of post-harvest land use restriction; and,
- Proximity to facilities for storage, transport or processing of plant material for subsequent analyses.

The resulting CFT data should be accepted as relevant by regulators in multiple geographies because of the science-based selection of trial sites in relevant agro-climatic zones, as well as satisfaction of local information and data requirements for ERA. By using this approach, the extended temporal and spatial distribution of CFTs additionally provides a “best case” for identifying any differences between the GE event and its comparator.

*Scenario II: A GE event that has been approved for cultivation in one country using data generated in local CFTs but which will be submitted for approval for cultivation in other countries in the future.*

As mentioned above, the first criterion is to check that the existing trials have been conducted and reported to meet the standards in the countries intended for future commercialization. Given acceptable pre-existing CFT data, the first step in this scenario is to map the locations of the existing CFT trials and then apply the framework to identify which, if any, of the agro-climatic zones where CFTs have already been conducted are representative of the agro-climatic zones where the event may be cultivated in other countries in the future. If the remote CFT sites are determined to be agro-climatic analogues, the product developer can then evaluate the potential scope of any additional CFTs which might be necessary to satisfy the needs of regulators in additional countries or regions. If additional trials are required to test risk hypotheses relevant to the ERA, then the considerations described under Scenario I will also apply and any remaining trial locations can be considered in the context of the ultimate countries of cultivation.

*Scenario III: A GE event that has been approved for cultivation in multiple countries.*

There are many examples of GE events that have already been evaluated in CFTs across a diversity of agro-climatic zones that encompass the range where the crop can be cultivated (*e.g*., MON15985 insect resistant cotton which has been authorized for cultivation in Australia, Brazil, Burkina Faso, India, Mexico, South Africa and the United States). In situations where applications for cultivation approval in new countries continue to be submitted, the product developer can use the framework approach to demonstrate that the accumulated body of evidence from both CFTs and commercial cultivation is sufficient to inform any future ERA. In this case, CFT data will have been collected from a large number of representative agro-climatic zones and under a wide range of agronomic production regimes experiencing many annual weather conditions and consequently no additional CFTs should be necessary unless new risk hypotheses have been developed.

## Discussion and recommended way forward

CFTs are regarded as an essential activity in the development of a GE plant intended for commercial cultivation, including for the generation of data to address risk hypotheses relevant to ERA. However, because CFTs are highly regulated they are also resource intensive, and so it is both a logistical and financial challenge to implement a CFT testing program at multiple sites and in multiple countries. This situation is exacerbated when CFT data submitted in support of an ERA are only considered to be relevant if generated in the local country, even when data are already available to address local risk hypotheses (or even prescriptive information requirements). While there may be cases where there is a legitimate, hypothesis-driven reason for CFTs to be undertaken on a site-specific basis, for most familiar crop species there is already an extensive body of peer-reviewed literature and data, as well as practical experience with crop breeding and cultivation, to support the concept of transportability of field trial data. Data from CFTs are particularly amenable to transportability because the trials are designed to be comparative (GE and non-GE in randomized plots at the same trial site), and are managed to control abiotic and biotic stressors that might confound the measurement of endpoints.

The conceptual framework for data transportability is fundamentally quite simple: the characteristics of the physical environment at trial sites can be used to demonstrate that a remote site has a local agro-climatic analogue. As long as the remote CFTs are designed, managed and reported in a manner that meets minimum local regulatory requirements, then the logic and process described here can be used to provide an evidence-based rationale for accepting trial data from a remote site as relevant to the local ERA (e.g., Scenarios II and III). In such cases, effective problem formulation will help determine whether sufficient, geographically-relevant data from remote CFTs exist to complete a risk characterization or if additional CFTs may be required to address local risk hypotheses. The conceptual framework can also be applied proactively to strategically identify CFT locations that will best represent the range of agro-climatic zones where a specific GE event is anticipated to be cultivated (e.g., Scenarios I and II).

The Working Group recognizes that the application of the framework will be more easily achieved if CFT design and reporting are standardized, and consequently recommends that an international expert body (e.g., the OECD Working Group on Harmonization of Regulatory Oversight in Biotechnology) consider the feasibility of developing guidance on this topic. In addition, the Working Group identified that the key variables for characterizing CFTs are climatic. Several existing peer-reviewed schemes have been reviewed for identifying global agro-climatic zones—either crop specific or for general agricultural production. These schemes have unique characteristics but several appear to meet the requirements for characterizing similarities between cropping regions in different countries. Nevertheless, before adopting one of these schemes in its current form, a careful examination of how well the current agro-climatic zone delineations work for the purposes of data transportability needs to be conducted. This will require close consultation with experts in agro-climatic zone approaches and the data sets that underlie these. Key factors for these discipline experts to evaluate might include:

- Whether agro-climatic zones based on crop-specific inputs provide substantially different results at the local country level compared with those derived from data identifying agricultural areas producing crops;
- Whether simpler zonation systems which address global crop coverage using fewer zones might be sufficient for the application of the conceptual framework as well as (and with increased simplicity) the more complex ones proposed here;
- Whether the processes used for global zonation are equally applicable for supporting regional data transportability or whether additional regional zonation would be beneficial;
- Whether the use of generalized, globally accepted agro-climatic zones to support CFT data transportability introduces unwarranted uncertainties compared to case-specific methods.

A transparent and peer-reviewed study of this nature will further support a robust, evidence-based rationale for accepting data from CFTs conducted in a remote country as suitable and potentially sufficient for local ERAs.

As proposed here, application of the conceptual framework for transportability of CFT data for ERA of GE plants should prove highly attractive and beneficial to both product developers and regulatory authorities. It promotes a strategic approach to identifying CFT site locations so that data accrued will be relevant to local and remote receiving environments and hence transportable from one regulatory jurisdiction to another. In some cases, this approach may extend both temporal and spatial distribution of trial sites which will ensure a potential “best case” for identifying any differences between the GE event and its appropriate counterpart. The conceptual framework additionally provides a scientifically defensible process for evaluating if existing CFT data from remote sites are relevant and/or sufficient for local ERAs. In both prospective and retrospective cases, unnecessary CFTs will be avoided which will decrease costs through more efficient application of human, institutional and financial resources. This will be particularly beneficial to public sector product developers and small enterprises that develop innovative GE events but cannot afford to replicate redundant CFTs.
